# An X‐ray micro‐CT protocol enables non‐destructive 3D visualization of glandular architecture in archived paraffin‐embedded prostate cancer tissue

**DOI:** 10.1002/2056-4538.70119

**Published:** 2026-10-13

**Authors:** Santiago Laguna‐Castro, Erna Mäkinen, Teemu Tolonen, Antti Kaipia, Jari Hyttinen

**Affiliations:** ^1^ Computational Biophysics and Imaging Group, Faculty of Medicine and Health Technology Tampere University Tampere Finland; ^2^ Department of Pathology Fimlab Laboratories Tampere Finland; ^3^ Department of Urology Tampere University Hospital Tampere Finland

**Keywords:** micro‐CT, X‐ray, 3D‐histology, paraffin‐embedded tissue, glandular architecture, prostate cancer, denoising, segmentation

## Abstract

The prostate is organized as a complex glandular network, and prostate cancer is accompanied by architectural changes that cannot always be inferred from two‐dimensional (2D) histology. The study of these features in three dimensions (3D) in cancerous tissue is important because glandular features have shown potential value for disease characterization and risk stratification beyond that obtained from individual 2D sections. Large collections of clinically annotated material preserved in paraffin remain an important research source. However, many 3D imaging methods require destructive or labor‐intensive preparation, limiting access to valuable material that needs to be re‐archived. In this proof‐of‐concept study, we developed an X‐ray micro‐computed tomography (micro‐CT) workflow for direct 3D imaging of archived paraffin‐embedded prostate specimens with near‐zero sample manipulation. Imaging and denoising parameters were optimized to balance image quality with acquisition time. Paraffin‐embedded prostate core punches (*n =* 5) from five radical prostatectomy specimens were imaged by micro‐CT and subsequently processed for histology. Digitized histological sections were aligned with corresponding micro‐CT planes for qualitative comparison, while pre‐sectioning micro‐CT data provided an intact geometric reference for exploratory assessment of histology‐associated dimensional changes at the whole‐core and glandular‐lumen levels. The optimized workflow yielded micro‐CT images with discernible glandular morphology and qualitative structural correspondence with matched histological sections. Exploratory measurements suggested modest dimensional change at the whole‐core level and greater variability at the lumen level; none of the tested deformation metrics reached conventional statistical significance. Illustrative 3D segmentation presented the glandular epithelium and lumina as spatially continuous structures within a cancer‐containing region. These findings support the feasibility of X‐ray micro‐CT as a non‐destructive approach for 3D characterization of prostate glandular architecture directly from paraffin‐embedded tissue, providing volumetric context for structures identified on histology while preserving the specimen for subsequent histological assessment.

## Introduction

The prostate is organized as a three‐dimensional (3D) network of glandular acini and ducts. Prostate cancer (PCa) development is accompanied by remodeling of this architecture, and the resulting glandular growth patterns underpin histopathological grading [1, 2, 3, 4, 5]. However, interpretation of these patterns in individual two‐dimensional (2D) sections can be difficult and variable [6, 7, 8, 9]. This limitation has motivated the development of 3D histological approaches [10, 11, 12], which have shown that features such as gland separation, fusion, branching, and luminal continuity may be difficult to infer from individual sections because their appearance depends on the sectioning plane [13, 14, 15, 16, 17, 18, 19].

Serial‐section reconstruction, confocal microscopy, and open‐top light‐sheet (OTLS) microscopy have been used to characterize prostate cancer architecture in 3D [12, 13, 14, 15, 16, 17, 18, 19, 20, 21, 22, 23, 24, 25, 26, 27]. These approaches provide different combinations of imaging volume and structural detail. Serial‐section reconstruction requires extensive sectioning and registration, whereas optical 3D workflows applied to FFPE tissue generally require deparaffinization, labeling, clearing, or other protocol‐specific preparation [14, 15, 16, 19, 20, 21, 22, 23, 24, 25, 28, 29]. These requirements may alter tissue geometry or consume material [16, 28, 29, 30, 31], which is particularly relevant because pathology archives and biobanks contain large, valuable FFPE collections whose availability for research may be constrained [32]. X‐ray micro‐computed tomography (micro‐CT) offers non‐destructive volumetric imaging [33, 34, 35, 36] and has been applied to paraffin‐embedded tissues [34, 35, 37, 38, 39, 40, 41, 42, 43], including prostate samples [37]. However, the weak native X‐ray attenuation contrast between soft tissue and paraffin makes direct visualization challenging, and contrast‐enhancing stains are frequently used in soft‐tissue micro‐CT [34, 35, 36, 41, 42, 44, 45, 46]. Application‐specific optimization is therefore needed to visualize glandular architecture directly in intact FFPE prostate tissue without additional contrast staining while maintaining compatibility with subsequent histological analysis [35, 37, 40].

Building on this rationale, we developed and optimized an X‐ray micro‐CT workflow for volumetric imaging of intact formalin‐fixed paraffin‐embedded (FFPE) prostate core punches retrieved from archived radical prostatectomy specimens, without additional contrast staining and with minimal manipulation after core retrieval. The workflow combined optimization of imaging conditions and denoising with alignment of the intact micro‐CT volumes to corresponding histological sections. We evaluated qualitative structural correspondence, identified histology‐associated artifacts, and performed an exploratory assessment of histology‐associated dimensional change using the pre‐sectioning micro‐CT data as an intact 3D geometric reference. We also demonstrated the technical feasibility of volumetric segmentation by visualizing an illustrative cancer‐containing glandular region in 3D. Together, these analyses were intended to assess the feasibility of micro‐CT as a non‐destructive method for characterizing glandular architecture in archived FFPE prostate tissue while retaining the same material for subsequent histological assessment or re‐archiving.

## Materials and methods

### Ethics statement and patient consent

FFPE radical prostatectomy specimens from patients with prostate cancer were obtained from Tampere Biobank (Tampere, Finland). The use of formalin‐fixed, paraffin‐embedded human prostate tissue was conducted in accordance with the Declaration of Helsinki (https://www.wma.net/policies‐post/wma‐declaration‐of‐helsinki/) guidelines and applicable legislation and institutional guidelines under Tampere Biobank's governance and was approved under ethical approval code R23018. Written informed consent was obtained from the patients for research use of their archived tissue samples and associated data under Tampere Biobank governance.

### Sample collection

The overall experimental workflow is summarized in Figure 1. A total of five prostates (*n =* 5) were included in this study. One paraffin core per specimen was retrieved using a 2‐mm‐diameter dermal biopsy punch. Each collected core targeted a region representative of a selected Gleason pattern or benign tissue. The sampled regions comprised Gleason patterns 3, 4, and 5 and two benign regions (Table 1).

**Figure 1:**
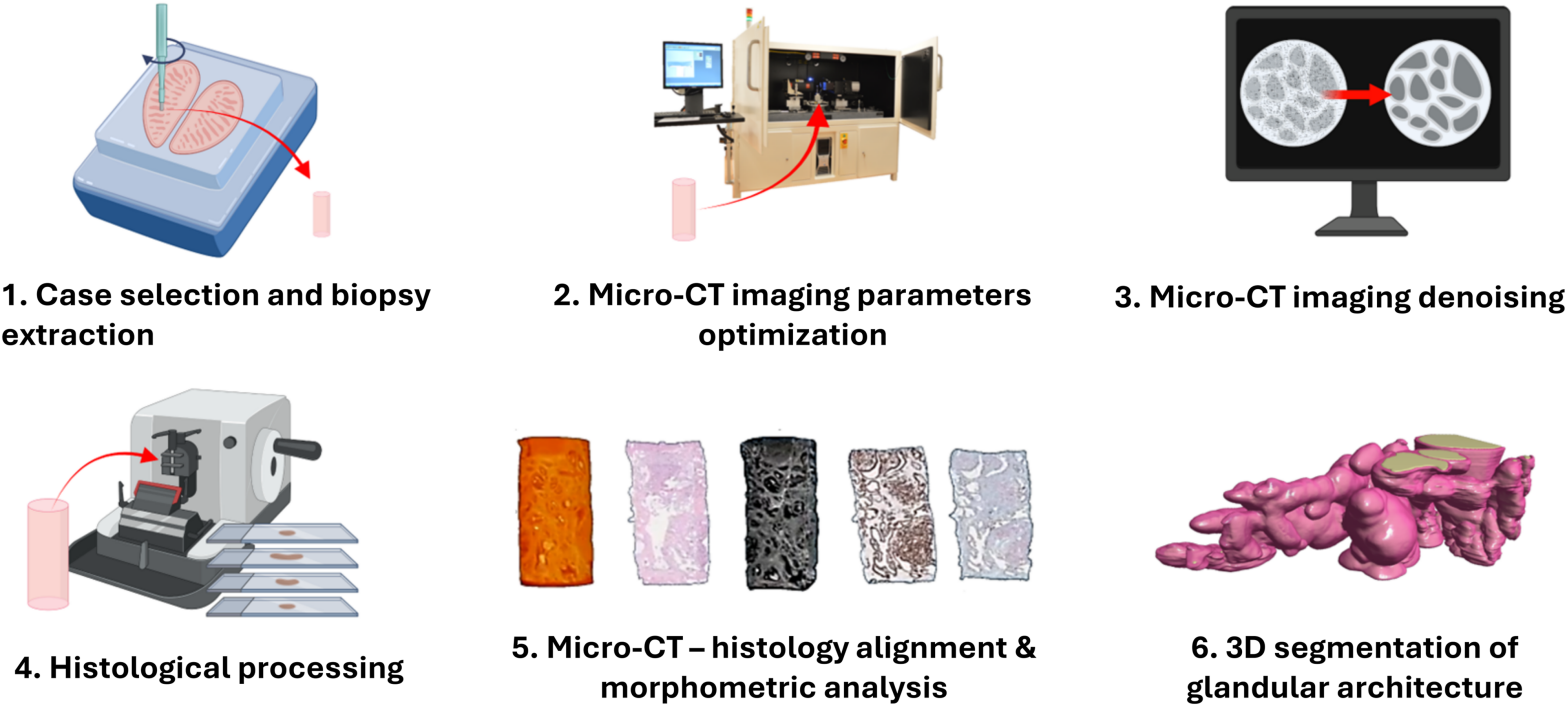
Experimental workflow for micro‐CT imaging of formalin‐fixed paraffin‐embedded prostate core punches retrieved from archived radical prostatectomy specimens. The workflow comprised case selection and core extraction, optimization of micro‐CT acquisition parameters, denoising of reconstructed micro‐CT images and subsequent histology. Histological slices and micro‐CT virtual slices are matched for qualitative and quantitative comparison. Illustrative segmentation of a glandular region demonstrates the feasibility of this approach to reveal the glandular architectures in 3D. Created in Biorender (2026). https://Biorender.com/h6t9b06

**Table 1:** List of samples included in the study and their main histological composition

Prostate core ID	Histological composition
1	Gleason pattern 3
2	Gleason pattern 5
3	Benign (atrophy + inflammation)
4	Gleason pattern 3 and benign tissue
5	Gleason pattern 3 and 4

### Micro‐CT system and imaging settings

Micro‐CT imaging was performed using a commercial MicroXCT‐400 system (Xradia, Inc.; now Carl Zeiss X‐ray Microscopy, Inc., Dublin, CA, USA). The acquisition geometry comprised a 120 mm source‐to‐object distance and a 13 mm sample‐to‐detector distance, and scans were acquired without beam filtration. Owing to this acquisition geometry, the images exhibited absorption‐based contrast and edge enhancement consistent with weak phase‐contrast effects. Prior to scanning, paraffin cores were transferred into 1‐ml syringes without additional processing or staining and mounted directly onto the micro‐CT sample holder (Figure 2). Imaging was performed using a 10× optical objective and 2× detector binning, yielding an isotropic voxel size of 2.47 μm. Each core was scanned over 360° using 1,600 projections with one frame per projection. To reduce ring artifacts, the sample was translated along the rotation axis during acquisition using the manufacturer's dynamic ring‐reduction mode. Tomographic volumes were reconstructed using XMReconstructor software (version 8.1.6599; Xradia, Inc./Carl Zeiss X‐ray Microscopy, Inc.) without beam‐hardening correction and saved as 3D TIFF images.

**Figure 2:**
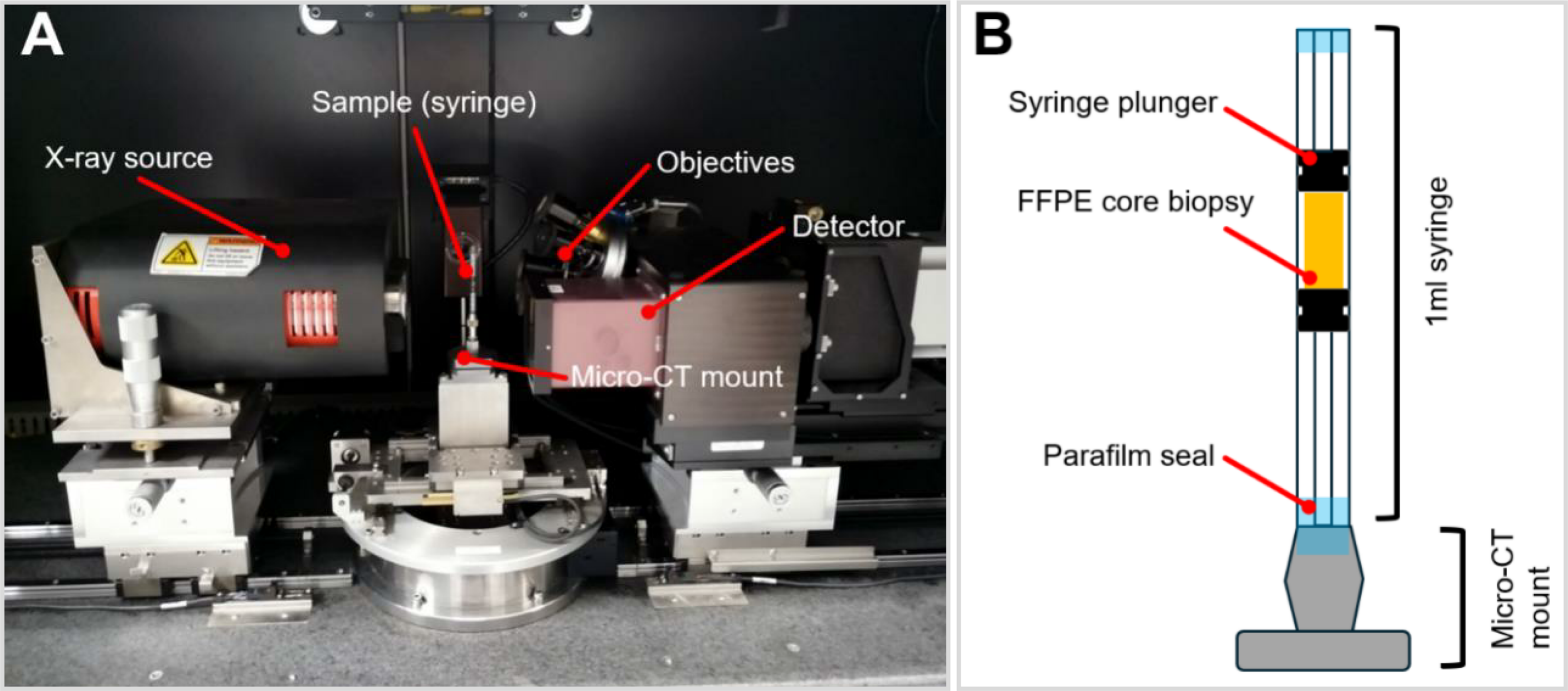
Micro‐CT imaging setup. (A) FFPE prostate core punch biopsy mounted inside the micro‐CT system between the X‐ray source and detector. (B) Schematic of the FFPE prostate core inside a 1‐ml syringe, coupled to the micro‐CT mount for tomographic imaging. No contrast staining was applied before scanning.

### Optimization of micro‐CT imaging parameters

Imaging parameters were optimized using one core as a pilot sample (sample 1; Table 1) to balance total scanning time and image quality. The optimized parameters were the X‐ray tube voltage and exposure time per tomographic projection.

#### X‐ray tube voltage

Serial acquisitions of the pilot core were performed at 40, 50, 60, and 80 kV to identify the tube voltage that maximized contrast between paraffin and embedded tissue. For each acquisition, contrast‐to‐noise ratio (CNR) values were calculated on Avizo 3D™ (version 2025.1, Thermo Fisher Scientific, Hillsboro, OR, USA). The same paired regions of interest were used across all scans, with one ROI placed in a paraffin‐only area and the other in an adjacent tissue‐containing region within the same core. These regions were used to calculate the CNR values. The tube voltage yielding the highest CNR was considered optimal; 50 kV was selected for subsequent exposure time optimization.

#### Exposure time

The pilot core was scanned with an exposure time of 75 s per projection, and this acquisition served as a high‐quality reference image. The same sample was re‐scanned at 50 and 25 s. The operational exposure time was defined as the shortest tested acquisition that still allowed consistent identification of gland boundaries, luminal spaces, and gland‐stroma interfaces by a board‐certified uropathologist. Based on these criteria, operational exposure time was set to 25 s.

### Denoising of low‐exposure micro‐CT scans

To mitigate noise associated with the low‐exposure acquisition, seven denoising methods were evaluated on the 25‐s pilot dataset, using the 75‐s exposure dataset as a baseline. Before denoising, the 25‐ and 75‐s volumes were rigidly co‐registered on Dragonfly™ (version 2025.1, Comet Technologies Canada Inc., Montréal, QC, Canada).

The evaluated denoising approaches included Gaussian [47], median [48], anisotropic diffusion [49], Kuwahara [50], bilateral [51] and non‐local means filtering [52], as well as a U‐Net‐based autoencoder [53]. Conventional filtering methods were implemented in Dragonfly™, except for non‐local means filtering, which was performed in Avizo 3D™.

The U‐Net‐based autoencoder was trained in Dragonfly™ as a 2D regression model using virtual sections extracted from the pilot 25‐s exposure volume. The same 25‐s sections were used as both input and reconstruction target, following a self‐reconstruction strategy intended to reflect a practical setting in which matched high‐exposure reference scans are not available for routine training. The resulting output was evaluated against the co‐registered 75‐s exposure dataset as an external higher‐exposure reference. Training was performed on 128 × 128 × 1 patches with a batch size of 32, network depth of 5, and initial filter count of 64. Optimization used the Adadelta algorithm with mean squared error (MSE) loss, and 20% of the training data was reserved for validation. The model was trained for up to 100 epochs with early stopping and learning‐rate reduction on plateau. Detailed parameter settings of each tested denoising algorithm are summarized in supplementary material, Table S1.

### Assessment of denoising performance

Denoising performance was quantified by comparing each denoised 25‐s dataset with the co‐registered 75‐s reference volume using the structural similarity index (SSIM) [54, 55], normalized root‐mean‐square error (NRMSE) [56], and peak signal‐to‐noise ratio (PSNR) [55]. SSIM was used to assess the preservation of structural information, whereas NRMSE and PSNR quantified overall voxel‐wise agreement with the reference image. Qualitative performance was evaluated by visual review of the denoised images, with final ratings determined by consensus among investigators with expertise in micro‐CT image interpretation. This evaluation considered noise reduction relative to the high‐exposure reference scan together with the preservation of gland boundaries and stromal texture. Each feature was scored separately for every denoising method using a scale from very good to very poor. Based on the combined quantitative and qualitative evaluation, anisotropic diffusion was selected as the preferred denoising method.

### Histological processing and slide digitization

All samples (*n =* 5) were imaged using the optimized micro‐CT protocol and subsequently processed for histology. The cores were re‐embedded in bullet‐shaped paraffin molds with their long axes oriented parallel to the block face and serially sectioned at 2.5 μm using an automatic rotary microtome. Sections from the top, middle, and bottom regions of each core were stained with hematoxylin and eosin (H&E), yielding a total of 42 sections. Two additional sections per core were prepared for BASA‐AMACR (Ventana Medical Systems, Inc., Tucson, AZ, USA) and pan‐cytokeratin (Pan‐CK; AE1/AE3/PCK26; Ventana Medical Systems, Inc.) immunohistochemistry to support the identification of glandular architecture in the H&E sections and corresponding micro‐CT images. All slides were digitized at 20× using a NanoZoomer S60 slide scanner (Hamamatsu Photonics K.K., Hamamatsu, Shizuoka, Japan) at a resolution of 442 nm/pixel in NDPI format and also exported as TIFF files for alignment with the micro‐CT data.

### Alignment of micro‐CT with histology

To enable side‐by‐side comparison of corresponding micro‐CT and histological planes, micro‐CT volumes from all cores were manually aligned with the digitized histological sections in Avizo™. Gross anatomical landmarks, including core geometry and tissue contours, were first used to establish the overall orientation of each 3D micro‐CT volume relative to the histological sections. Each histological section was then matched to its corresponding micro‐CT plane by interactively adjusting the plane depth and angle until visual correspondence between shared morphological features was achieved.

### Histology‐associated tissue deformation assessment

Two types of linear measurements were obtained from matched histological and micro‐CT images for each sample: core‐level distances measured between anatomical landmarks near the tissue margins and glandular lumen diameters measured in selected luminal cross‐sections. Intact micro‐CT images were treated as the pre‐sectioning geometric reference, as FFPE cores remain physically intact during micro‐CT imaging. In both analyses, measurements were resolved along two orientations defined relative to the microtome slicing direction: perpendicular and parallel to the cutting plane (Figure 5A,B). For clarity, these directions are referred to as the X and Y axes, respectively. Histological measurements were performed in NDP.view2™ (version 2.8.24.0; Hamamatsu Photonics K.K.) and replicated on the corresponding micro‐CT virtual sections in Fiji [57] using identical endpoint criteria.

#### Core‐level measurements

For each histological section, two linear distances were measured between reproducible anatomical landmarks near the tissue margins. The same distances were measured on the corresponding micro‐CT virtual slice (Figure 5A). Across the 42 sections analyzed, this yielded 84 paired histology–micro‐CT comparisons, corresponding to 168 individual measurements.

#### Lumen cross‐section measurements

Local deformation was assessed by measuring lumen diameters in glandular profiles identified in aligned histological sections and their corresponding micro‐CT planes. Five profiles per core were selected from eligible luminal structures with clearly delineated contours and good visual correspondence between the two modalities (*n =* 5 cores; total *n =* 25; Figure 5B). For each selected profile, two perpendicular lumen diameters were measured using the same *X*/*Y* convention as that applied in the core‐level analysis.

#### Tissue distortion metrics

For both global and local measurements, deformation was expressed as the percentage change in histology relative to micro‐CT in each direction:

$$\%{∆}_{i}=100\times \frac{\;\left({\text{histology}}_{i}-\text{micro}-{\mathrm{CT}}_{i}\right)}{\left(\text{micro}-{\mathrm{CT}}_{i}\right)},\;i\in \left\{X,Y\right\}$$

where i denotes the measurement direction, with X perpendicular to the slicing direction and Y parallel to it. Positive values indicate larger measurements on histology than on micro‐CT, whereas negative values indicate smaller measurements in histology than on micro‐CT.

Directional asymmetry was summarized using a directional difference term (*D*):

D=%ΔY−%ΔX

Positive *D* values indicate that the percentage change in Y is numerically greater than that in X, whereas negative values indicate the opposite.

#### Statistical analyses

Because multiple measurements were obtained from each core, all deformation outcomes were summarized at the core level, and each core was treated as the unit of analysis (*n =* 5). For the core‐level analysis, %Δ*X*, %Δ*Y*, and *D* were calculated for each matched histology‐micro‐CT section pair and averaged within each core. For the lumen‐level analysis, the same metrics were calculated for each selected lumen profile and averaged within each core. The primary analysis was descriptive, with per‐core values summarized as mean ± SD for both measurement scales. Directional and scale‐dependent patterns were interpreted on an exploratory basis from the per‐core estimates and graphical comparisons. As a secondary exploratory analysis, two‐sided exact one‐sample Wilcoxon signed‐rank tests against zero were performed for %Δ*X*, %Δ*Y*, and *D* at both scales, with *α* = 0.05. Analyses were performed using IBM SPSS Statistics (version 30.0.0.0; IBM Corp., Armonk, NY, USA).

To assess intra‐observer repeatability, 25 whole‐core and all 25 gland‐level measurements were repeated by the same observer at the same targets. Repeatability of %Δ*X*, %Δ*Y*, and *D* was evaluated using single‐measure intraclass correlation coefficients (ICCs) from a two‐way mixed‐effects model with absolute agreement and 95% confidence intervals.

### Qualitative assessment of artifacts caused by histological processing

Histological sections were visually compared with their corresponding micro‐CT planes to assess the added value of micro‐CT in preserving tissue morphology. The evaluation focused on histology‐associated alterations not evident in the pre‐sectioning micro‐CT data, including distortion or loss of recognizable microscopic structures and sectioning‐related artifacts.

### Three‐dimensional segmentation and visualization of cancer‐containing regions

To demonstrate the volumetric utility of the optimized micro‐CT workflow, an illustrative glandular region from the core containing predominant Gleason pattern 3 (sample 1) was segmented using the reconstructed 3D data in Avizo™. The segmentation aimed to delineate luminal spaces and glandular epithelium and combined manual annotation, volumetric interpolation between annotated planes, surface smoothing, and manual correction. Corresponding H&E and immunohistochemical sections were used to support the identification of glandular structures. Three‐dimensional renderings were then generated to visualize glandular morphology and spatial organization within the intact biopsy core.

## Results

### Micro‐CT imaging optimization

#### Tube voltage selection

CNR between paraffin and embedded prostate tissue was evaluated at 40, 50, 60, and 80 kV. The highest CNR was obtained at 50 kV (8.76), compared with values of 8.41 at 40 kV, 8.21 at 60 kV, and 6.97 at 80 kV (Figure 3A). Accordingly, 50 kV was selected for subsequent imaging.

**Figure 3:**
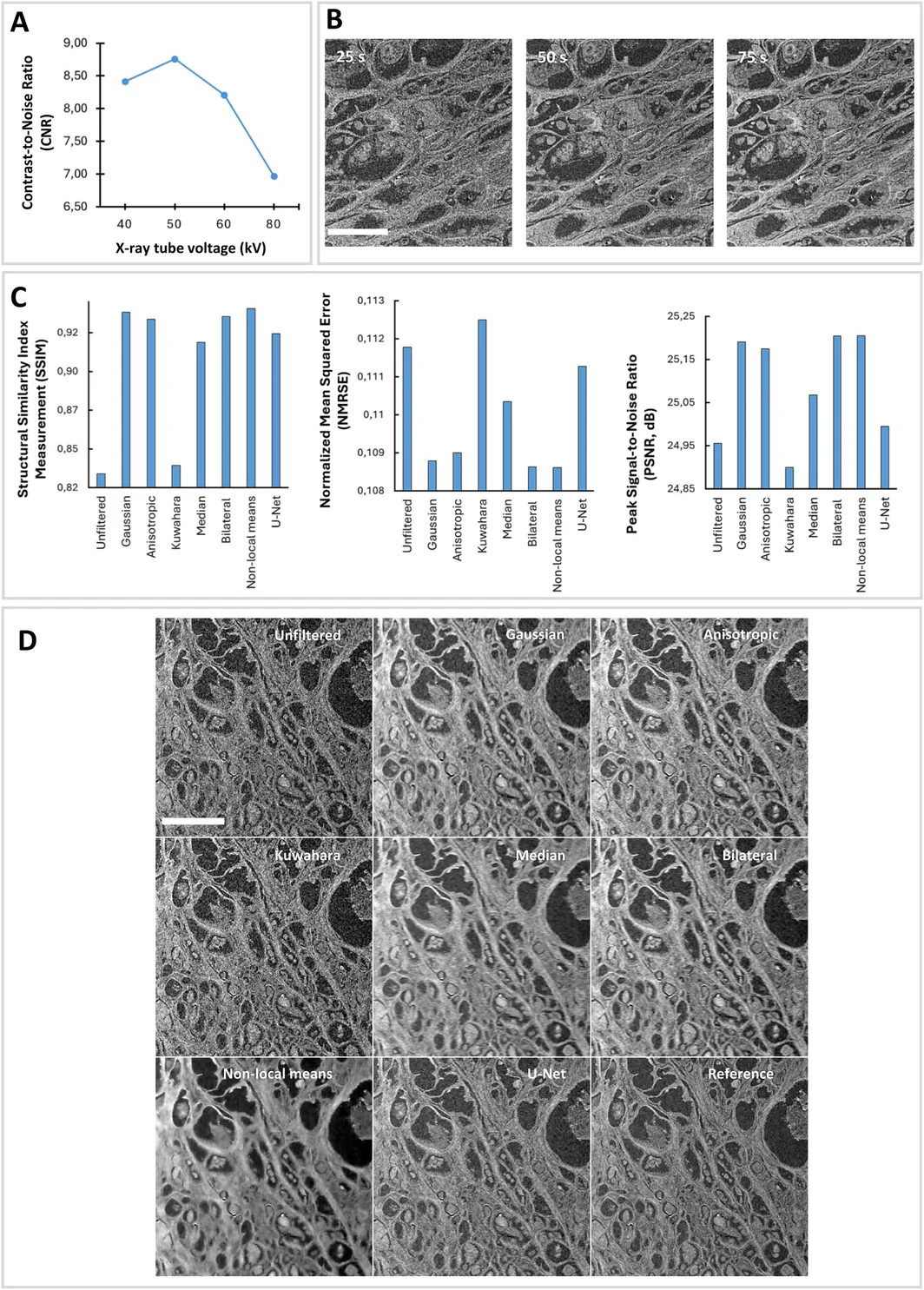
Optimization of micro‐CT acquisition parameters and denoising performance. (A) Contrast‐to‐noise ratio (CNR) measured at tube voltages of 40, 50, 60, and 80 kV in the pilot prostate core. The highest CNR was obtained at 50 kV. (B) Representative region of the sample imaged at exposure times of 25, 50, and 75 s per projection. The 25‐s acquisition preserved interpretable glandular architecture while reducing total scan duration from approximately 33 to 11 h. (C) Quantitative comparison of denoising performance using the structural similarity index (SSIM), normalized root‐mean‐square error (NRMSE), and peak signal‐to‐noise ratio (PSNR) relative to the 75‐s reference scan. (D) Visual comparison of denoising methods applied to the 25‐s acquisition. Scale bars: (B) 400 μm; (D) 500 μm.

#### Exposure time selection

Exposure time per projection was evaluated at 25, 50, and 75 s. Although longer exposure times reduced image noise, gland boundaries, luminal spaces, and gland‐stroma interfaces remained discernible at 25 s (Figure 3B).

At 1,600 projections, total scan durations were approximately 11 h at 25 s, 22 h at 50 s, and 33 h at 75 s. Because the 25‐s acquisition preserved relevant morphology while substantially reducing scan time, it was selected as the operational exposure setting for all subsequent datasets.

#### Denoising performance and filter selection

In the pilot sample, the unfiltered 25‐s acquisition showed baseline values of SSIM = 0.829, NRMSE = 0.112, and PSNR = 24.96 dB when compared with the corresponding 75‐s reference (Figure 3C). All tested denoising methods improved these metrics. Non‐local means filtering achieved the highest overall similarity to the reference, with SSIM = 0.936, NRMSE = 0.109, and PSNR = 25.21 dB. Comparable results were obtained with bilateral filtering (SSIM = 0.930, NRMSE = 0.109, PSNR = 25.20 dB) and anisotropic diffusion (SSIM = 0.929, NRMSE = 0.109, PSNR = 25.17 dB). Overall, quantitative differences among the top‐performing methods were minimal (Figure 3C).

Denoised images were qualitatively compared with the high‐exposure reference to visually assess noise reduction, preservation of gland boundaries, and maintenance of stromal texture (Figure 3D; Table 2). Among the evaluated methods, anisotropic diffusion provided the most balanced visual performance, combining good preservation of gland boundaries with very good maintenance of stromal texture, despite a slight softening of local structural detail. By comparison, the remaining conventional denoising methods tended to over‐smooth the images, leading to a loss of fine tissue detail. The U‐Net autoencoder closely reproduced the noise level and tissue boundaries of the 75‐s reference. However, small morphological inconsistencies were observed, indicating the limited reproducibility of this approach. Based on the combined quantitative and qualitative assessment, anisotropic diffusion was selected and applied to all datasets for subsequent analyses.

**Table 2:** Qualitative assessment of denoising methods for the pilot dataset acquired with an exposure time of 25 s per projection, using the 75 s exposure scan as reference

Method	Noise reduction	Glandular boundary preservation	Texture preservation	Main limitations
Unfiltered	N/A	Moderate‐poor	Very poor	Structural details are visible, but noise limits interpretability
Gaussian	Good	Good	Poor	Some small structural details are smoothed out
Median	Good	Moderate‐good	Poor‐moderate	General over‐smoothing of texture and significant loss of fine structural detail
Kuwahara	Poor	Poor	Poor	Does not provide any major improvement compared to the unfiltered image
Anisotropic diffusion	Good‐moderate	Good	Very good	Slight reduction in local structure contrast
Bilateral filtering	Good	Very good	Good	General over‐smoothing of texture, very strong loss of fine structural detail
Non‐local means	Very good	Good	Very poor	General over‐smoothing of texture, almost complete of fine structural detail
U‐Net autoencoder	Good‐moderate	Very good	Moderate	Replicates the noise level, texture and gland boundaries of the reference image but does not consistently replicate fine‐detail structures

### Histology – micro‐CT structural correspondence

All histological sections were identified within their corresponding micro‐CT volumes and aligned, enabling direct comparison between H&E and immunostained sections from different biopsy levels and the corresponding micro‐CT virtual sections (Figure 4A,B). Across all cores, paired micro‐CT‐histology images showed qualitative agreement in tissue contours and distribution of gland‐rich and stromal regions. Recurrent features such as epithelial contours, glandular lumina, luminal concretions, and fibromuscular stroma were recognizable in both imaging modalities (Figure 4B). Despite the overall structural correspondence, localized discrepancies between histology and micro‐CT were also observed (Figure 4C). These differences were consistent with artifacts caused by histological processing, including tissue loss, tearing, and expansion of the histological slices.

**Figure 4:**
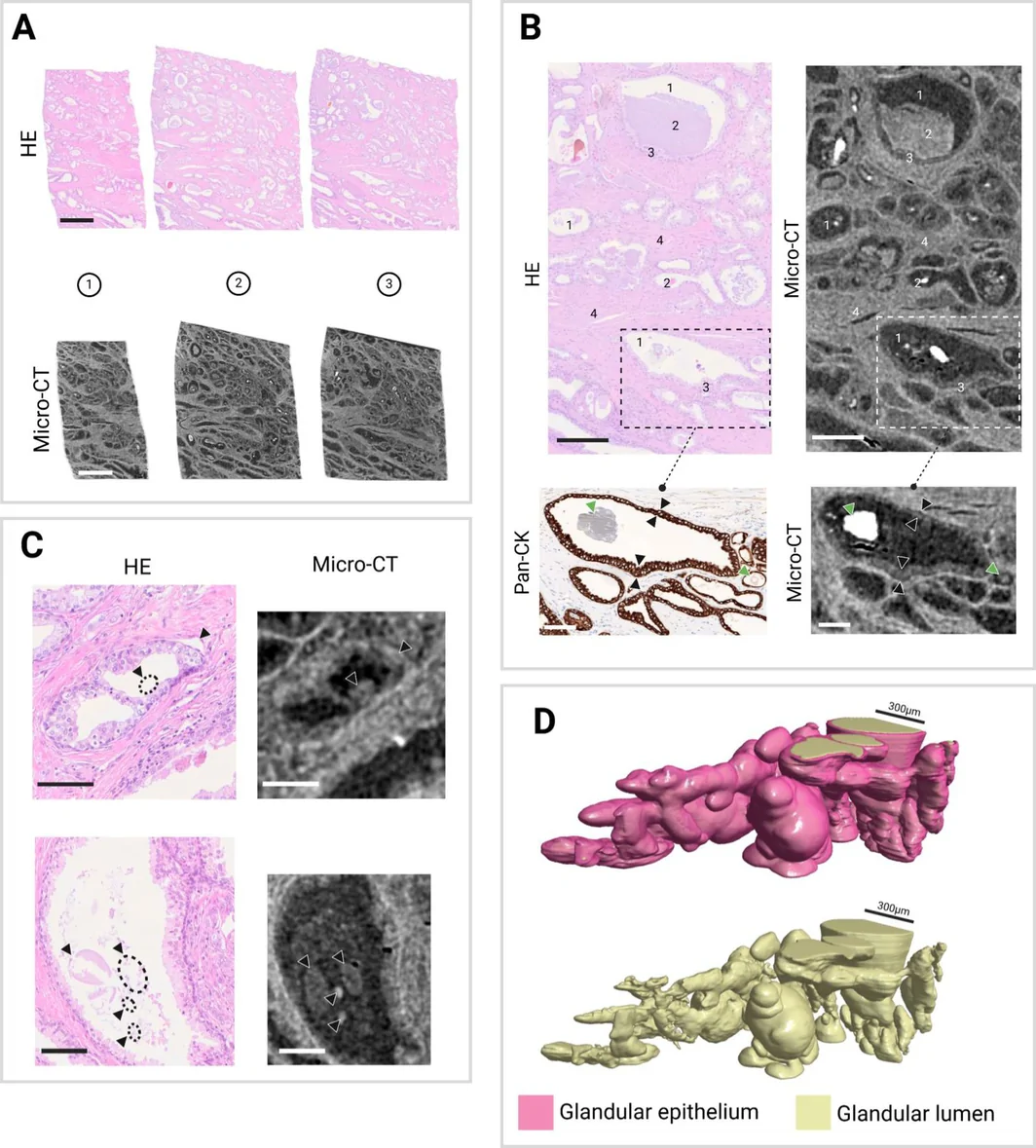
Structural correspondence between histological sections and matched micro‐CT images, examples of histology‐associated artifacts, and an illustrative 3D segmentation of prostatic glandular architecture. (A) Representative correspondence between hematoxylin and eosin (H&E) sections (top row) and matched micro‐CT virtual sections (bottom row) across three depths (1–3) of a paraffin‐embedded prostate core. (B) Matched intermediate magnification H&E and micro‐CT views showing corresponding glandular structures in both modalities. Labels 1–4 indicate (1) glandular lumen, (2) intraluminal concretions, (3) glandular epithelium, and (4) adjacent fibromuscular stroma. Boxed regions are shown below at higher magnification in an adjacent pan‐cytokeratin (Pan‐CK) section and the corresponding micro‐CT view. Black arrowheads indicate glandular epithelium and luminal contours. Green arrowheads indicate intraluminal concretions with different levels of attenuation in the micro‐CT image. (C) Additional high‐magnification matched H&E and micro‐CT examples showing histology‐associated artifacts, including tissue tearing, the absence of intraluminal concretions on histology, and dimensional differences between modalities. (D) Manual 3D segmentation of a glandular region in the micro‐CT volume acquired from the core containing predominant Gleason pattern 3 (sample 1). Glandular epithelium is shown in pink, and glandular lumina are shown in yellow. Scale bars: (A) 500 μm; (B) top row 200 μm and bottom row 90 μm; (C) 90 μm.

### Three‐dimensional visualization of segmented glandular architecture

The micro‐CT dataset from sample 1, which contained tissue representative of Gleason pattern 3, enabled manual 3D segmentation of glandular structures, with separate rendering of glandular epithelium and luminal spaces (Figure 4D). The segmented volume showed that glandular profiles visible in individual virtual sections were part of continuous structures extending through the tissue depth. Composite renderings clarified the spatial relationship between the epithelial compartment and the enclosed luminal spaces, whereas isolated lumen renderings highlighted local luminal continuity and branching. Together, these reconstructions provided volumetric architectural context that complemented the matched 2D histological sections and illustrated the potential of micro‐CT for the 3D assessment of glandular organization.

### Quantification of histology‐induced tissue deformation

#### Core‐level deformation

At the core level, dimensional changes were modest (Figure 5C; Table 3). On average, biopsy‐core dimensions increased by 5.23 ± 3.01% perpendicular to the histological slicing direction (%Δ*X*) and decreased by 1.73 ± 6.57% parallel to the slicing direction (%Δ*Y*) relative to micro‐CT. The mean directional difference was *D* = −6.95 ± 5.49, a lower dimensional change parallel to the sectioning direction than perpendicular to it.

**Figure 5:**
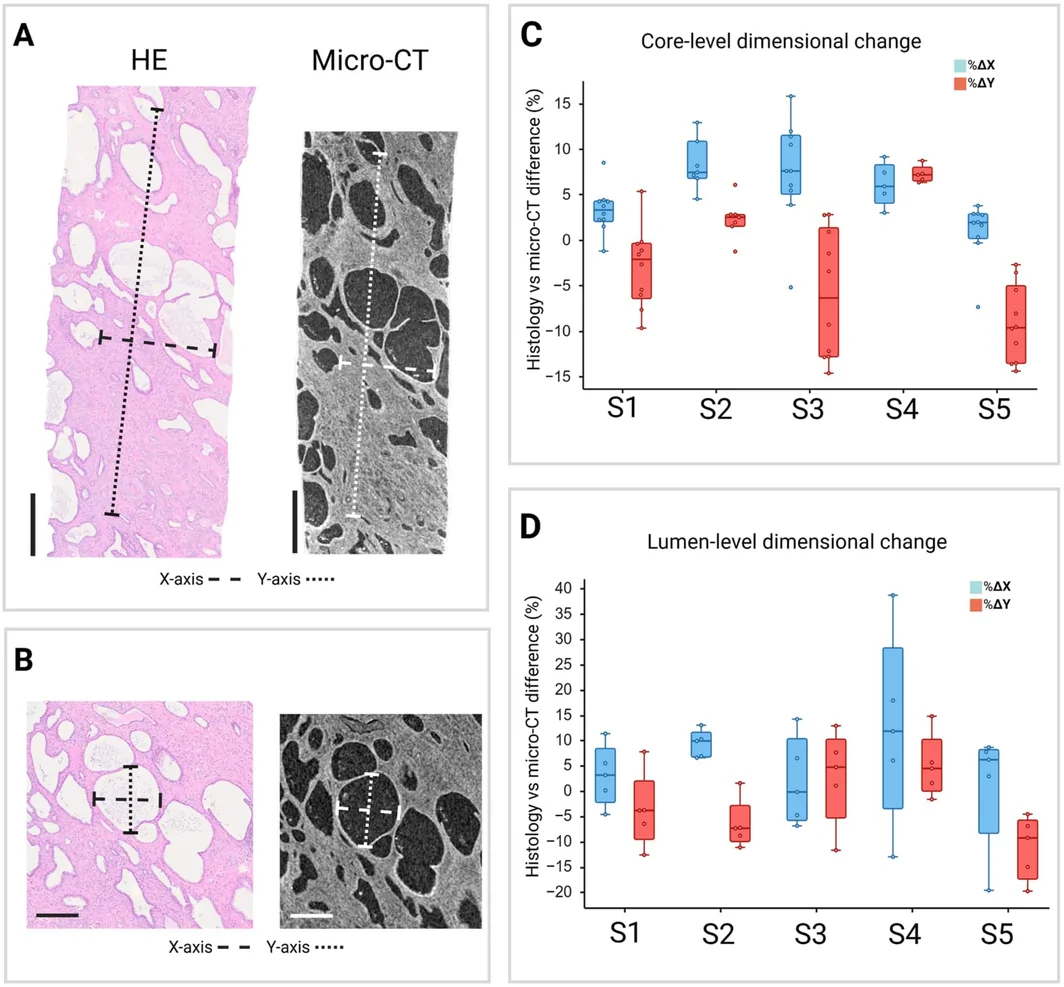
Assessment of dimensional change in histology relative to micro‐CT at whole‐core and glandular lumen levels. (A) Representative matched hematoxylin and eosin (H&E) and micro‐CT images illustrating the core‐level measurement scheme. Two perpendicular dimensions were measured in the histological image and in the corresponding micro‐CT plane: the *X*‐axis (dashed line), perpendicular to the histological slicing direction, and the *Y*‐axis (dotted line), parallel to it. (B) Representative matched H&E and micro‐CT images showing the glandular lumen measurement scheme, using the same axis convention. (C) Whole‐core dimensional change for each sample (S1–S5), expressed as the percentage change in histology relative to micro‐CT in the *X* and *Y* directions. (D) Lumen‐level dimensional change for each sample (S1–S5), also expressed as change in histology relative to micro‐CT in the *X* and *Y* directions. In panels (C) and (D), boxes indicate the interquartile range with the median shown as a horizontal line; whiskers extend to 1.5× the interquartile range, and individual measurements are overlaid as dots. Scale bars: (A) 500 μm; (B) 250 μm.

**Table 3:** Dimensional change in histology relative to micro‐CT and intra‐observer repeatability at whole‐core and glandular‐lumen levels

Scale	Metric	Primary analysis		Intra‐observer repeatability analysis	
		Mean ± SD %	*p* *	ICC	95% CI^†^
Whole‐core biopsy	%Δ*X*	5.23 ± 3.01	0.0625	0.807	0.614–0.909
	%Δ*Y*	−1.73 ± 6.57	0.6250	0.985	0.966–0.993
	*D* = %Δ*Y* − %Δ*X*	−6.95 ± 5.49	0.1250	0.897	0.783–0.953
Glandular lumen	%Δ*X*	5.54 ± 4.98	0.0625	0.906	0.800–0.957
	%Δ*Y*	−2.72 ± 6.67	0.4375	0.906	0.799–0.958
	*D* = %Δ*Y* − %Δ*X*	−8.26 ± 6.44	0.1250	0.860	0.711–0.935

%Δ*X* and %Δ*Y* represent percentage dimensional change in histology relative to micro‐CT, measured perpendicular and parallel to the slicing direction, respectively. Positive values indicate larger measurements in histology than in micro‐CT, whereas negative values indicate smaller measurements in histology than in micro‐CT. *D* denotes the directional difference (%Δ*Y* − %Δ*X*). The *p* values were obtained using two‐sided exact one‐sample Wilcoxon signed‐rank tests against 0 based on per‐core summaries (*n =* 5).

* Statistical significance was defined as *p* < 0.05.

^†^ Intra‐observer repeatability was assessed using 25 repeated measurements at each measurement scale; ICCs are single‐measure, absolute‐agreement coefficients from a two‐way mixed‐effects model, with 95% confidence intervals.

Because only five cores were included, conventional statistical significance could not be achieved with the exact two‐sided Wilcoxon signed‐rank test; the analyses were therefore considered exploratory. The corresponding exact *p* values were 0.0625 for %Δ*X*, 0.6250 for %Δ*Y*, and 0.1250 for *D*, and none reached conventional statistical significance.

#### Lumen‐level deformation

At the lumen level, deformation estimates were greater in magnitude and variability than at the whole‐core level in this cohort (Figure 5D; Table 3). On average, lumen dimensions increased by 5.54 ± 4.98% perpendicular to the slicing direction (%Δ*X*) and decreased by 2.72 ± 6.67% parallel to the slicing direction (%Δ*Y*) relative to micro‐CT. The mean directional difference was *D* = −8.26 ± 6.44 percentage points, reflecting a lower dimensional change parallel to the sectioning direction than perpendicular to it. Exact two‐sided Wilcoxon signed‐rank tests yielded *p* = 0.0625 for %Δ*X*, *p* = 0.4375 for %Δ*Y*, and *p* = 0.1250 for *D*; none reached conventional statistical significance.

#### Intra‐observer measurement repeatability

Intra‐observer repeatability was high for all deformation measurements. ICCs ranged from 0.807 to 0.985 at the whole‐core level and from 0.860 to 0.906 at the glandular‐lumen level (Table 3).

## Discussion

This proof‐of‐concept study demonstrates that X‐ray micro‐CT can resolve 3D prostate glandular architecture from intact FFPE core punches with minimal manipulation. Reconstructed volumes showed qualitative correspondence with matched histological sections and provided an intact geometric reference for exploratory histology‐associated deformation of slices. Final measurements suggested greater local variability at the lumen than at the whole‐core level, without statistical significance. Nevertheless, tissue expansion and deformation during routine histological processing are well documented [30, 58, 59, 60]. Technical demonstration of the 3D segmentation showed that glandular epithelium and lumina could be followed through tissue depth. The overall workflow remained compatible with subsequent histology and immunohistochemistry.

Serial‐section reconstruction showed that apparently separate Gleason pattern 3 glands can form interconnected tubular networks and demonstrated spatial continuity between pattern 3 and poorly formed pattern 4 [13, 14]. The 3D confocal studies further characterized prostate cancer growth patterns, identifying interconnected tubular architectures encompassing Gleason pattern 3 and several pattern 4 and 5 morphologies, distinct from the contiguous epithelial architecture of cribriform pattern 4 and solid pattern 5 [15, 17]. OTLS microscopy has extended this work toward quantitative analysis, with 3D glandular features contributing to benign‐malignant discrimination and showing greater value than corresponding 2D features for prostate cancer risk stratification [22, 23, 27]. Optical coherence tomography (OCT) additionally enabled rapid visualization of prostate microarchitecture in fresh tissue, highlighting a complementary route toward clinical imaging [61].

These 3D imaging methods involve different trade‐offs in structural detail, sample processing, and workflow burden. Serial‐section reconstruction retains conventional histological detail but consumes tissue and requires extensive sectioning, digitization, and alignment of slices [13, 14, 16]. Confocal microscopy and OTLS can provide finer cellular and molecular information [15, 20, 21, 22] compared to micro‐CT, but their workflows usually require deparaffinization, tissue clearing, and labeling. These steps can take from several hours to multiple days [15, 20, 24] and may induce tissue distortion, protein loss, lipid removal, or reduced antigen preservation [28, 31]. Our micro‐CT protocol circumvents these alterations through direct imaging of the FFPE sample, with minimal pre‐imaging processing. Micro‐CT 11 h per core acquisition time is relatively long if compared with the acquisition times of the aforementioned 3D imaging methods. However, these extensive times are offset by the minimal sample preparation required.

Costs are facility‐dependent and mainly reflect operator and instrument time. Relative to preparation‐intensive workflows, micro‐CT may shift a greater proportion of costs from sample preparation and consumables toward scanner access and instrument time. Access to micro‐CT may be limited by the need for dedicated instrumentation, although centralized imaging facilities can provide this capability and FFPE material is relatively easy to transport.

Beyond these practical considerations, the methodological contribution lies in obtaining isotropic whole‐core 3D information directly from intact archival FFPE prostate tissue despite its weak native X‐ray contrast, while preserving the specimen for subsequent histology. This capability is particularly relevant to archival research, as pathology departments and biobanks contain large collections of FFPE specimens [32], but research access to diagnostic blocks can be constrained by the need to preserve this material for future clinical use [62].

The study has several limitations. With a small cohort (*n =* 5), deformation measurements are strictly exploratory. Accordingly, the architectural features shown should not be generalized to specific Gleason patterns. Histology‐to‐micro‐CT plane matching was done manually, which introduces some degree of procedural uncertainty. Nevertheless, measurement repeatability assessment showed good intra‐observer repeatability. No beam‐hardening correction or dedicated sensitivity analysis was performed. Although no conspicuous artifacts were observed and lumen measurements were based on visible morphological boundaries rather than fixed intensity thresholds, subtle effects on apparent boundaries cannot be excluded. Downstream molecular analysis after X‐ray imaging of FFPE material was not part of this study. However, previous work demonstrated preserved DNA and RNA integrity and successful mutation testing after synchrotron‐based X‐ray imaging of human FFPE samples [63]. Laboratory micro‐CT has also been reported to preserve RNA integrity and gene‐expression profiles in human tissue [64]. Together, these studies support the feasibility of combining X‐ray‐based 3D imaging with subsequent molecular analysis, but direct validation with the present protocol is required.

Future work should focus on larger cohorts, systematic architectural 3D mapping, automated segmentation, improved registration, shorter acquisition protocols, and direct validation of downstream molecular compatibility. In conclusion, X‐ray micro‐CT provides non‐destructive 3D access to glandular architecture directly in archived FFPE prostate tissue without additional contrast staining. The same specimen remains available for conventional histology, allowing volumetric features to be related to subsequent histopathological findings. This creates a complementary opportunity to investigate prostate cancer architecture using material from pathology archives and biobanks.

## Author contributions statement

SL‐C contributed to the conceptualization, methodology, investigation, formal analysis, visualization, writing of the original draft, and review and editing of the manuscript. EM contributed to the investigation, resources, and review and editing of the manuscript. TT contributed to the conceptualization, resources, and supervision of the study. AK contributed to the conceptualization, resources, supervision, and review and editing of the manuscript. JH contributed to the conceptualization, resources, supervision, and review and editing of the manuscript.

## Supporting information


**Figure S1.** Micro‐CT virtual sections and matched hematoxylin and eosin histological slices of all FFPE prostate punch core biopsies included in the study
**Table S1.** Denoising methods and parameter settings evaluated for micro‐CT image optimization
**Table S2.** Absolute paired histology and micro‐CT measurements at whole‐core level
**Table S3.** Absolute paired histology and micro‐CT subset of repeated measurements at the whole‐core level for the intra observer repeatability measurement evaluation
**Table S4.** Absolute paired histology and micro‐CT measurements at the glandular‐lumen level
**Table S5.** Absolute paired histology and micro‐CT repeated measurements at the glandular‐lumen level for intra‐observer repeatability evaluation

**Movie S1.** Illustrative comparison of micro‐CT virtual sections with matched HE histological sections from an FFPE prostate core punch

**Movie S2.** Visualization of the 3D segmentation of an illustrative glandular network region included in the study

**Movie S3.** Sequential micro‐CT virtual sections showing intraluminal structures with strong differences in X‐ray attenuation values

## Data Availability

The datasets generated and analyzed during the current study are available from the corresponding author on reasonable requests, subject to institutional, ethical, and data protection restrictions governing archived human tissue imaging data.
