# Supplementary material for: An X‐ray micro‐CT protocol enables non‐destructive 3D visualization of glandular architecture in archived paraffin‐embedded prostate cancer tissue
Source: J Pathol Clin Res. 2026 Oct 13;12(6):e70119. doi: 10.1002/2056-4538.70119 (PMC13659285; doi:10.1002/2056-4538.70119)
Supplement: Supplementary file 1 [file CJP2-12-e70119-s002.pdf]

An X-ray micro-CT protocol enables non-destructive 3D visualization of glandular architecture in archived paraffin-embedded prostate cancer tissue

S Laguna-Castro *et al*. *J Pathol Clin Res*. https://doi.org/10.1002/2056-4538.70119

Supplementary Figure S1
Supplementary Tables S1–S5

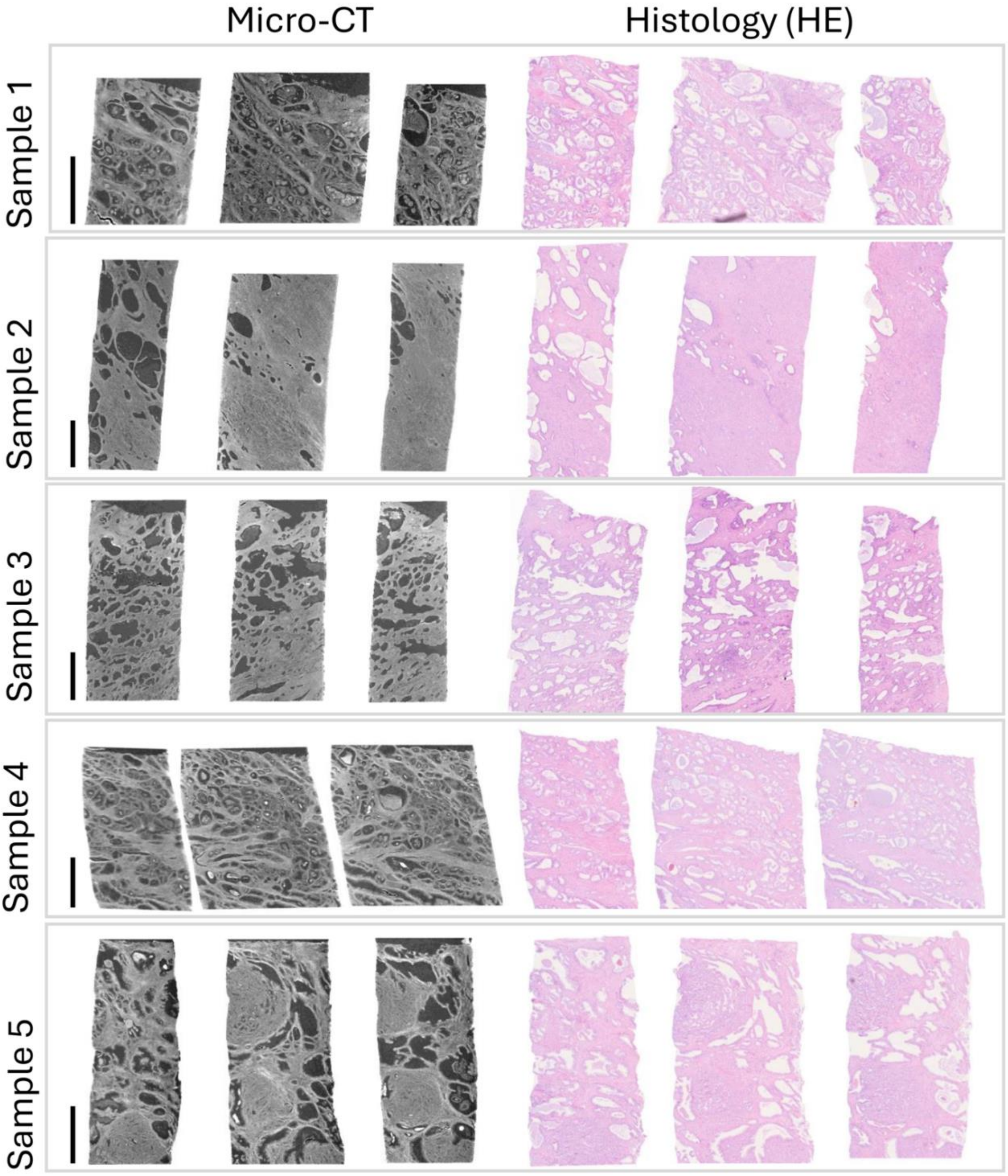

Figure S1. Micro-CT virtual sections and matched hematoxylin and eosin histological slices of all FFPE prostate punch core biopsies included in the study. Sections are representative of top, middle and bottom regions of the cores. Scale bars, 1000 µm

Table S1. Denoising methods and parameter settings evaluated for micro-CT image optimization.

**Method**	**Mode**	**Parameter settings**
Gaussian filter	3D	σ = 1.5; kernel size = 9
Bilateral filter	2D	kernel size = 9; σ color = 1000; σ spatial = 20
Kuwahara filter	2D	kernel size = 3
Anisotropic diffusion	2D	iterations = 5; kappa= 0.05
Median filter	3D	kernel size = 9
Non-local means	2D	spatial SD = 5; intensity SD = 0.9; search window = 9 px; patch size = 10 px
U-Net (autoencoder)	2D	Architecture: depth level = 5; initial filter count = 64. Training: batch size = 32; epochs = 100; patch size = [128,128,1]; loss function = mean squared error (MSE); optimization algorithm = Adadelta; learning rate = 1.0; learning rate scheduler = Reduce LR on plateau; validation split = 20%

Table S2. Absolute paired histology and micro-CT measurements at whole-core level. D, directional deformation difference, calculated as %ΔY − %ΔX.

Sample ID	Slice ID	X- axis measurements (µm)		Y-axis measurements (µm)		X histology – X micro-CT	Y histology – Y micro-CT	ΔX (%)	ΔY (%)	D
		Micro-CT	Histology	Micro-CT	Histology					
1	1	726.23	757.00	1859.99	1680.00	30.78	–179.99	4.24	–9.68	–13.91
	2	997.77	1040.00	1500.31	1410.00	42.23	–90.31	4.23	–6.02	–10.25
	3	708.59	735.00	1606.61	1600.00	26.41	–6.61	3.73	–0.41	–4.14
	4	1341.53	1380.00	1072.10	1070.00	38.47	–2.10	2.87	–0.20	–3.06
	5	1187.70	1240.00	1171.26	1140.00	52.30	–31.26	4.40	–2.67	–7.07
	6	1262.84	1370.00	1451.09	1340.00	107.16	–111.09	8.49	–7.66	–16.14
	7	1320.26	1350.00	1798.46	1700.00	29.74	–98.46	2.25	–5.47	–7.73
	8	966.67	988.00	1537.83	1620.00	21.33	82.17	2.21	5.34	3.14
	9	1123.68	1110.00	1557.90	1540.00	–13.68	–17.90	–1.22	–1.15	0.07
	10	1034.62	1050.00	1290.45	1270.00	15.38	–20.45	1.49	–1.58	–3.07
2	11	841.03	898.00	3200.28	3290.00	56.97	89.72	6.77	2.80	–3.97
	12	1195.31	1280.00	2315.47	2380.00	84.69	64.53	7.09	2.79	–4.30
	13	1321.79	1420.00	3447.94	3500.00	98.21	52.06	7.43	1.51	–5.92
	14	1762.68	1990.00	3488.53	3700.00	227.32	211.47	12.90	6.06	–6.83

	15	1608.85	1740.00	3316.29	3380.00	131.15	63.71	8.15	1.92	–6.23
	16	1398.27	1550.00	2916.65	2880.00	151.73	–36.65	10.85	–1.26	–12.11
	17	1062.06	1110.00	2741.36	2810.00	47.94	68.64	4.51	2.50	–2.01
3	18	914.38	1010.00	2667.06	2740.00	95.62	72.94	10.46	2.73	–7.72
	19	1100.28	1160.00	3171.44	3260.00	59.72	88.56	5.43	2.79	–2.64
	20	1217.73	1410.00	3417.40	3300.00	192.27	–117.40	15.79	–3.44	–19.22
	21	1481.05	1570.00	2565.40	2190.00	88.95	–375.40	6.01	–14.63	–20.64
	22	1706.17	1900.00	2973.58	3000.00	193.83	26.42	11.36	0.89	–10.47
	23	1429.31	1600.00	2953.85	2910.00	170.69	–43.85	11.94	–1.48	–13.43
	24	1292.03	1390.00	2667.87	2420.00	97.97	–247.87	7.58	–9.29	–16.87
	25	1049.60	1090.00	2847.68	2500.00	40.40	–347.68	3.85	–12.21	–16.06
	26	865.38	931.00	2270.96	1980.00	65.62	–290.96	7.58	–12.81	–20.39
	27	505.27	479.00	1629.84	1420.00	–26.27	–209.84	–5.20	–12.87	–7.68
4	28	989.62	1080.00	1817.73	1950.00	90.38	132.27	9.13	7.28	–1.86
	29	657.26	696.00	1693.07	1800.00	38.74	106.93	5.89	6.32	0.42
	30	1106.92	1140.00	1214.26	1320.00	33.08	105.74	2.99	8.71	5.72
	31	1103.68	1160.00	1409.16	1510.00	56.32	100.84	5.10	7.16	2.05
	32	1387.21	1490.00	1471.96	1570.00	102.79	98.04	7.41	6.66	–0.75

5	33	923.76	942.00	2499.33	2410.00	18.24	–89.33	1.97	–3.57	–5.55
	34	1040.04	1070.00	1827.43	1680.00	29.96	–147.43	2.88	–8.07	–10.95
	35	1344.57	1370.00	3090.33	2740.00	25.43	–350.33	1.89	–11.34	–13.23
	36	1190.70	1210.00	2814.93	2660.00	19.30	–154.93	1.62	–5.50	–7.12
	37	1565.07	1570.00	2581.00	2330.00	4.93	–251.00	0.32	–9.72	–10.04
	38	1551.59	1610.00	3148.89	2720.00	58.41	–428.89	3.76	–13.62	–17.38
	39	914.78	940.00	2949.96	2870.00	25.22	–79.96	2.76	–2.71	–5.47
	40	1001.19	1030.00	3352.91	2870.00	28.81	–482.91	2.88	–14.40	–17.28
	41	1183.79	1180.00	3179.81	2750.00	–3.79	–429.81	–0.32	–13.52	–13.20
	42	560.25	519.00	435.60	394.00	–41.25	–41.60	–7.36	–9.55	–2.19

Table S3. Absolute paired histology and micro-CT subset of repeated measurements at the whole-core level for the intra observer repeatability measurement evaluation. D, directional deformation difference, calculated as %ΔY − %ΔX.

Sample ID	Slice ID	X- axis measurements (μm)		Y-axis measurements (μm)		$X_{histology} - X_{micro\text{-}CT}$	$Y_{histology} - Y_{micro\text{-}CT}$	ΔX (%)	ΔY(%)	D
		Micro-CT	Histology	Micro-CT	Histology					
1	1	726.30	766.00	1852.73	1690.00	39.70	−162.73	5.47	−8.78	−14.25
	3	710.01	737.00	1596.88	1600.00	26.99	3.12	3.80	0.20	−3.61
	5	1187.77	1230.00	1173.73	1130.00	42.23	−43.73	3.56	−3.73	−7.28
	6	1261.75	1380.00	1495.07	1330.00	118.26	−165.07	9.37	−11.04	−20.41
	9	1121.06	1120.00	1524.28	1540.00	−1.06	15.72	−0.09	1.03	1.13
2	11	835.00	892.00	3199.15	3300.00	57.00	100.85	6.83	3.15	−3.67
	13	1304.52	1410.00	3448.85	3510.00	105.48	61.15	8.09	1.77	−6.31
	15	1604.03	1740.00	3308.23	3390.00	135.97	81.77	8.48	2.47	−6.01
	16	1416.58	1550.00	2913.33	2870.00	133.42	−43.33	9.42	−1.49	−10.91
	17	1001.53	1110.00	2756.01	2820.00	108.47	63.99	10.83	2.32	−8.51
3	19	1098.35	1160.00	3196.99	3250.00	61.65	53.01	5.61	1.66	−3.95
	21	1477.13	1560.00	2561.11	2180.00	82.87	−381.11	5.61	−14.88	−20.49
	22	1706.58	1920.00	2969.65	2990.00	213.42	20.35	12.51	0.69	−11.82
	23	1411.97	1580.00	2955.59	2900.00	168.04	−55.59	11.90	−1.88	−13.78
	25	1039.50	1070.00	2852.86	2500.00	30.50	−352.86	2.93	−12.37	−15.30
4	28	1005.71	1070.00	1815.97	1950.00	64.29	134.03	6.39	7.38	0.99
	29	660.70	699.00	1712.64	1810.00	38.30	97.37	5.80	5.69	−0.11
	30	1132.41	1160.00	1230.01	1320.00	27.59	89.99	2.44	7.32	4.88
	31	1100.21	1150.00	1407.65	1510.00	49.79	102.35	4.53	7.27	2.75
	32	1393.87	1500.00	1471.80	1560.00	106.13	88.20	7.61	5.99	−1.62
5	35	1353.87	1380.00	3094.92	2730.00	26.13	−364.92	1.93	−11.79	−13.72
	37	1584.70	1590.00	2600.46	2360.00	5.30	−240.46	0.33	−9.25	−9.58
	38	1557.98	1610.00	3117.80	2710.00	52.02	−407.80	3.34	−13.08	−16.42
	39	919.80	944.00	2933.28	2850.00	24.20	−83.28	2.63	−2.84	−5.47
	41	1169.14	1191.00	3169.64	2740.00	21.86	−429.64	1.87	−13.55	−15.42

Table S4. Absolute paired histology and micro-CT measurements at glandular-lumen level. D, directional deformation difference, calculated as %ΔY – %ΔX.

Sample ID	Gland ID	X- axis measurements (μm)		Y-axis measurements (μm)		$X_{histology} - X_{micro\text{-}CT}$	$Y_{histology} - Y_{micro\text{-}CT}$	ΔX (%)	ΔY(%)	D
		Micro-CT	Histology	Micro-CT	Histology					
1	1	264.21	252.00	135.81	127.00	–12.21	–8.81	–4.62	–6.49	–1.87
	2	157.79	158.00	141.43	136.00	0.21	–5.43	0.13	–3.84	–3.97
	3	84.69	94.30	44.45	47.90	9.61	3.45	11.35	7.77	–3.58
	4	267.30	282.00	187.06	180.00	14.71	–7.06	5.50	–3.77	–9.28
	5	234.58	242.00	130.46	114.00	7.42	–16.46	3.16	–12.62	–15.78
2	6	86.36	97.60	128.31	119.00	11.24	–9.30	13.02	–7.25	–20.27
	7	278.82	298.00	214.66	218.00	19.18	3.34	6.88	1.55	–5.33
	8	379.98	405.00	434.26	396.00	25.02	–38.26	6.58	–8.81	–15.40
	9	107.95	119.00	129.54	120.00	11.05	–9.54	10.24	–7.36	–17.60
	10	109.18	120.00	97.46	86.60	10.82	–10.86	9.91	–11.15	–21.05
3	11	133.24	133.00	185.06	187.00	–0.24	1.95	–0.18	1.05	1.23
	12	108.57	124.00	103.63	117.00	15.43	13.37	14.22	12.90	–1.32
	13	86.77	80.80	81.42	71.90	–5.97	–9.52	–6.88	–11.70	–4.82
	14	148.04	141.00	112.68	118.00	–7.04	5.32	–4.76	4.72	9.48
	15	231.94	247.00	218.37	235.00	15.06	16.64	6.50	7.62	1.12
4	16	72.17	85.10	66.62	69.60	12.93	2.98	17.91	4.47	–13.44
	17	48.73	54.50	46.26	47.00	5.77	0.74	11.84	1.59	–10.25
	18	24.37	33.80	34.23	39.30	9.43	5.07	38.72	14.79	–23.92
	19	157.50	167.00	90.47	89.00	9.50	–1.47	6.03	–1.63	–7.66
	20	81.84	71.20	100.34	106.00	–10.64	5.66	–13.00	5.64	18.64
5	21	364.35	375.00	271.42	259.00	10.65	–12.41	2.92	–4.57	–7.50
	22	262.37	285.00	157.09	126.00	22.63	–31.09	8.63	–19.79	–28.42
	23	519.80	552.00	278.82	253.00	32.20	–25.82	6.20	–9.26	–15.45
	24	303.49	327.00	152.98	130.00	23.51	–22.98	7.75	–15.02	–22.77
	25	160.50	129.00	382.52	356.00	–31.50	–26.52	–19.63	–6.93	12.69

Table S5. Absolute paired histology and micro-CT repeated measurements at the glandular-lumen level for the intra observer measurement repeatability evaluation. D, directional deformation difference, calculated as %ΔY − %ΔX.

Sample ID	Gland ID	X- axis measurements (μm)		Y-axis measurements (μm)		$X_{histology} - X_{micro\text{-}CT}$	$Y_{histology} - Y_{micro\text{-}CT}$	ΔX (%)	ΔY(%)	D
		Micro-CT	Histology	Micro-CT	Histology					
1	1	258.65	250.00	132.56	131.00	–8.65	–1.56	–3.35	–1.18	2.17
	2	153.42	159.00	140.75	139.00	5.58	–1.75	3.64	–1.24	–4.88
	3	83.64	95.10	45.68	46.30	11.46	0.62	13.70	1.36	–12.35
	4	269.15	286.00	187.05	176.00	16.85	–11.05	6.26	–5.91	–12.17
	5	235.81	241.00	132.10	114.00	5.19	–18.10	2.20	–13.70	–15.90
2	6	88.53	98.90	119.52	121.00	10.38	1.48	11.72	1.24	–10.48
	7	280.12	299.00	218.21	218.00	18.88	–0.21	6.74	–0.10	–6.84
	8	382.73	404.00	441.21	396.00	21.27	–45.21	5.56	–10.25	–15.80
	9	108.31	120.00	129.02	119.00	11.69	–10.02	10.80	–7.77	–18.56
	10	109.51	122.00	95.66	85.70	12.49	–9.96	11.41	–10.41	–21.82
3	11	133.05	132.00	185.27	186.00	–1.05	0.73	–0.79	0.40	1.19
	12	108.89	125.00	97.38	117.00	16.12	19.62	14.80	20.15	5.35
	13	86.68	81.50	80.25	71.80	–5.18	–8.45	–5.98	–10.53	–4.55
	14	142.69	144.00	113.82	122.00	1.31	8.18	0.92	7.19	6.27
	15	226.73	248.00	218.53	237.00	21.27	18.47	9.38	8.45	–0.93
4	16	78.41	85.20	63.58	72.50	6.79	8.92	8.66	14.02	5.36
	17	55.84	57.00	47.96	48.20	1.16	0.24	2.08	0.50	–1.58
	18	26.75	33.40	35.75	39.30	6.65	3.55	24.87	9.93	–14.94
	19	160.80	166.00	93.53	89.10	5.20	–4.43	3.23	–4.74	–7.97
	20	85.56	75.40	103.25	104.00	–10.16	0.75	–11.87	0.72	12.60
5	21	353.24	371.00	276.56	260.00	17.76	–16.56	5.03	–5.99	–11.01
	22	264.87	286.00	161.05	127.00	21.13	–34.05	7.98	–21.14	–29.12
	23	538.55	554.00	280.24	255.00	15.45	–25.24	2.87	–9.01	–11.88
	24	312.53	326.00	150.74	130.00	13.47	–20.74	4.31	–13.76	–18.07
	25	166.09	132.00	400.70	357.00	–34.09	–43.70	–20.52	–10.91	9.62
